# Real-world evidence of finerenone in Type 2 diabetes: addressing confounding and follow-up duration

**DOI:** 10.1093/ehjcvp/pvaf087

**Published:** 2025-12-16

**Authors:** Yi-Hsien Chen, Yu-Wei Fang, Ming-Hsien Tsai

**Affiliations:** Department of Family Medicine, Shin Kong Wu Ho-Su Memorial Hospital, Taipei 11101, Taiwan; Department of Internal Medicine, Shin Kong Wu Ho-Su Memorial Hospital, No. 95, Wenchang Rd, Shilin District, Taipei 11101, Taiwan; School of Medicine, Fu Jen Catholic University, New Taipei City 242062, Taiwan; Department of Internal Medicine, Shin Kong Wu Ho-Su Memorial Hospital, No. 95, Wenchang Rd, Shilin District, Taipei 11101, Taiwan; School of Medicine, Fu Jen Catholic University, New Taipei City 242062, Taiwan

We thank Dong and colleagues for their insightful interest in our article regarding the association between non-steroidal mineralocorticoid receptor antagonists (MRAs) and cardiovascular events in Type 2 diabetes,^1^ and for their letter outlining key methodological considerations.^2^ We appreciate the opportunity to clarify the context of our findings and discuss the future of real-world evidence (RWE) in this domain.

First, regarding the concerns about unmeasured confounders—specifically medication adherence and baseline left ventricular ejection fraction—we agree that these are inherent limitations of retrospective electronic health record analyses. Unlike randomized controlled trials that enforce strict adherence protocols to determine ‘efficacy’, our study aimed to evaluate ‘effectiveness’ in a real-world setting where adherence varies.^3^ To mitigate selection bias, we employed robust propensity score matching including distinct pharmacological proxies [e.g. use of renin–angiotensin system blockade, beta-blockers, and sodium-glucose co-transporter 2 (SGLT2) inhibitor], cardiovascular comorbidities, and laboratory values, which serve as surrogate markers for heart failure severity. While residual confounding is possible, the consistency of our results across sensitivity analyses suggests a genuine treatment effect.

Second, the authors noted that our follow-up of 24 months may be insufficient to capture long-term cardiovascular and renal outcomes. We respectfully submit that this duration is clinically relevant. The pivotal finerenone in reducing kidney failure and disease progression in diabetic kidney disease (FIDELIO-DKD) trial^4^ demonstrated significant benefits with a median follow-up of 2.6 years, which is comparable to our observation window. Our findings essentially provide an early real-world validation of the trends observed in FIDELIO-DKD, proving that the divergence in event rates begins relatively early. As finerenone is a newly approved drug, real-world follow-up is currently limited by its short time on the market. Future longitudinal studies will be vital to assess ‘legacy effects’ and long-term renal preservation as clinical experience matures.

Third, regarding the attenuated effect sizes observed in women and patients with preserved renal function, we agree this heterogeneity warrants further investigation. This observation may reflect reduced statistical power within smaller subgroups or a ‘ceiling effect’ where lower-risk populations demonstrate smaller absolute risk reductions. These findings align with the clinical imperative to prioritize high-risk phenotypes who derive the greatest absolute benefit from MRA therapy.^4,5^

Finally, concerning safety, we concur that monitoring discontinuation is vital. However, we highlight that our study explicitly analysed safety endpoints, demonstrating a significantly lower risk of hyperkalaemia with finerenone compared with spironolactone. This safety signal is a critical differentiator for non-steroidal MRAs and supports their use in routine practice.

In conclusion, we are grateful for the constructive dialogue initiated by Dong et al.^2^ Despite the limitations of retrospective data, we believe our study adds crucial RWE that complements pivotal clinical trials, supporting the early and safe initiation of finerenone in eligible patients with Type 2 diabetes.

## Data Availability

There was no new data created in this study.

## References

[1] Chen Y-H, Fang Y-W, Chen M-T, Liou H-H, Tsai M-H. Nonsteroidal mineralocorticoid receptor antagonists and cardiovascular events in type 2 diabetes: a retrospective study. Eur Heart J Cardiovasc Pharmacother 2025;11:610–619. 40503674 10.1093/ehjcvp/pvaf048 PMC12582653

[2] Dong W, Li D, Zhao J. Finerenone for diabetic hearts: a critical appraisal of MACE benefits and the research road ahead. Eur Heart J Cardiovasc Pharmacother 2025. 10.1093/ehjcvp/pvaf086 PMC12946958 41400296

[3] Sherman RE, Anderson SA, Dal Pan GJ, Gray GW, Gross T, Hunter NL, LaVange L, Marinac-Dabic D, Marks PW, Robb MA, Shuren J, Temple R, Woodcock J, Yue LQ, Califf RM. Real-world evidence—what is it and what can it tell us? N Engl J Med 2016;375:2293–2297. 27959688 10.1056/NEJMsb1609216

[4] Bakris GL, Agarwal R, Anker SD, Pitt B, Ruilope LM, Rossing P, Kolkhof P, Nowack C, Schloemer P, Joseph A, Filippatos G. Effect of finerenone on chronic kidney disease outcomes in type 2 diabetes. N Engl J Med 2020;383:2219–2229. 33264825 10.1056/NEJMoa2025845

[5] Pitt B, Filippatos G, Agarwal R, Anker SD, Bakris GL, Rossing P, Joseph A, Kolkhof P, Nowack C, Schloemer P, Ruilope LM. Cardiovascular events with finerenone in kidney disease and type 2 diabetes. N Engl J Med 2021;385:2252–2263. 34449181 10.1056/NEJMoa2110956

